# Polyvagal Theory: A biobehavioral journey to sociality

**DOI:** 10.1016/j.cpnec.2021.100069

**Published:** 2021-06-16

**Authors:** Stephen W. Porges

**Affiliations:** a Traumatic Stress Research Consortium at the Kinsey Institute, Indiana University, Bloomington, IN, 47405, USA; b Department of Psychiatry, University of North Carolina at Chapel Hill, Chapel Hill, NC, 27514, USA

**Keywords:** Polyvagal theory, Autonomic nervous system, Development, Evolution, Neuroception, Social behavior, Threat, Safety

## Abstract

A polyvagal perspective clarifies the neurobiological and biobehavioral shifts that occurred during evolutionary transition from asocial reptiles to social mammals. This transition enabled mammals, unlike their reptilian ancestors, to derive a biological benefit from social interactions. This innovation enabled social behavior to function as a neuromodulator that could efficiently regulate and optimize autonomic function to support homeostatic processes. This journey is highlighted by the phylogenetic transition during which the autonomic nervous system was repurposed to suppress defensive strategies to support and express sociality. The product of this transition was an autonomic nervous system with capacities to self-calm, to spontaneous socially engage others, and to mitigate threat reactions in ourselves and others through social cues. Thus, social behavior became embedded with specific neurobiological processes that had capabilities to support homeostatic functions leading to optimized health, growth, and restoration. Polyvagal Theory emphasizes sociality as the core process in mitigating threat reactions and supporting mental and physical health.

## Highlights

• Sociality in mammals co-evolved with a repurposed autonomic nervous system.
• Evolution repurposed the mammalian ventral vagal complex in the brainstem to support sociality.
• Social behavior functions as a neuromodulator optimizing behavioral, autonomic, and emotional state regulation.
• Neuroception reflexively detects risk or safety without awareness and shifts autonomic state to support adaptive behaviors.
• Unlike their reptilian ancestors, mammals have a neuroception to safety that fosters sociality by calming autonomic state.

## 1 The transdisciplinary origins of Polyvagal Theory

A polyvagal perspective clarifies the evolutionary transition that enabled mammals to be social and to use sociality as a mechanism to regulate and optimize physiological state and homeostatic processes. This journey is highlighted by the phylogenetic transition from reptiles to mammals, during which the autonomic nervous system was repurposed to suppress defensive strategies in order to support and express sociality. The product of this transition was an autonomic nervous system with capacities to self-calm, to spontaneous socially engage others, and to mitigate threat reactions in ourselves and others through social cues. Thus, social behavior became embedded with specific neurobiological processes that had capabilities to support homeostatic functions that would lead to optimized health, growth, and restoration. Succinctly, Polyvagal Theory emphasizes sociality as a core process in mitigating threat and supporting mental and physical health.

To understand the origins of Polyvagal Theory, visualize a Rubik's cube with surfaces representing different disciplines moving in time as each field is selectively updated by new information. Metaphorically, Polyvagal Theory is the solution of a Rubik's puzzle, a solution to how evolution repurposed the mammalian autonomic nervous system to contain defensive reactions and enable sociality to thrive. This metaphor is helpful, since Polyvagal Theory is a product of an extraction of principles derived from the integration of several disciplines each with its own history, research paradigms, literature, methodology, and theoretical context. The theory evolved to ask new questions and was not conceptualized to replace the predominant theories associated with these foundational disciplines. Thus, the solution to the puzzle produced a transdisciplinary theory that had a foundational basis but was not limited or biased by any specific discipline.

This paper is structured to explain how evolution and development of neuroanatomical structures and neurophysiological processes contribute to Polyvagal Theory. The initial assumptions of the Polyvagal Theory provide insights into deconstructing autonomic and behavioral reactions to threat consistent with the Jacksonian principle of dissolution or evolution in reverse [1]. Thus, evolution became the initial organizing principle for the theory. As the theory developed other disciplines provided convergent support of the hierarchy of autonomic states introduced in the initial publication of the Polyvagal Theory [2]. The study of development and maturation paralleled the insights extracted from evolutionary biology (see Ref. [3]. In addition, dissolution of autonomic state converged with the personal narratives of survivors of trauma (see Ref. [4]. The theory validated the immobilization shutdown responses reported by survivors.

All three pathways (i.e., evolution, development, clinical observation) help us understand the dependence of sociality on the neural regulation of the autonomic nervous system. From evolutionary biology, we see shifts in structure and function of the autonomic nervous system as asocial vertebrates evolved into social mammals with a biological imperative to connect, nurse, cooperate, and trust select others (e.g., Ref. [5]. From developmental biology, we observe shifts in neural regulation of the autonomic nervous system starting during the last trimester that prepare the newborn to suckle, breathe, and to co-regulate. From the study of trauma, we see the dissolution of many of these social systems with profound consequences for mental and physical health (see Ref. [4]. Similarly, during difficult deliveries, the neonate follows a predictable pattern of dissolution that is characterized by a sequence of loss of heart rate variability, metabolically costly tachycardia, and finally the shutting down of vital systems as indexed by life-threatening bradycardia (Reed et al., 1999).

## 2 Historical context

The early conceptualization of the vagus in mammals focused on an undifferentiated efferent (motor) pathway that was assumed to modulate tone concurrently to several target organs. Little attention was directed in afferent (sensory) limb of the vagus that provides dynamic feedback to the brainstem structures regulating the efferent outflow. Thus, neural circuits regulating the supradiaphragmatic (e.g., myelinated vagal pathways originating in the nucleus ambiguus and terminating primarily above the diaphragm) were not functionally distinguished from the subdiaphragmatic (e.g., unmyelinated vagal pathways originating in the dorsal motor nucleus of the vagus and terminating primarily below the diaphragm). Without this distinction, research and theory in psychophysiology and psychosomatic medicine focused on the paired antagonism between the parasympathetic and sympathetic innervation to target organs. The consequence of an emphasis on paired antagonism in physiology was an acceptance and use of global constructs (i.e., autonomic balance, sympathetic arousal, vagal tone) without documenting the specific neural pathways and feedback circuits involved in dynamically regulating the autonomic nervous system. Missing was an understanding of how areas of the brain communicated with and regulated the visceral end organs of the autonomic nervous system.

More than 50 years ago, W. Hess [6] proposed that the “autonomic” nervous system was not solely “vegetative” and automatic but was an integrated system with both peripheral and central neurons. Hess demonstrated the influence of the hypothalamus on the autonomic nervous system. By emphasizing the central mechanisms that mediate the dynamic regulation of peripheral organs, Hess anticipated the need for methodologies and technologies to continuously monitor the neural circuits involving both defined brain structures and peripheral nerves in the regulation of visceral function and state. Consistent with these insights, the Polyvagal Theory was proposed and methods were suggested to extract the time course of the influence of two vagal circuits on the beat-to-beat heart rate pattern [2,7].

Polyvagal Theory was introduced as an attempt to shift the science of psychophysiology from a descriptive science conducting empirical studies and describing correlations between psychological and physiological processes to an inferential science generating and testing hypotheses related to common neural pathways involving both mental and physiological processes. It was the first volley in a conceptual dialog challenging the questions and methods involved in psychophysiological research and especially in the sub-domain of cardiovascular psychophysiology.

Psychophysiology emerged in the 1960s, a relatively atheoretical empirical period of science in which a simplistic arousal theory dominated research. Basically, arousal theory emphasized that arousal was a linear construct indexing a dimension from low to high levels of activation that could be measured or inferred from observing behavior or physiology. The relationship between arousal and performance was often portrayed as an inverted U- shaped function in which optimal performance occurred within a midlevel range, while poor performance was observed at low and high levels of arousal. This relationship was known as the Yerkes-Dodson law [8]. Metaphorically, arousal represented the energy of the human nervous system. Arousal was easily understood, since when it was reflected behaviorally it could be quantified as greater activity and when reflected autonomically it could be observed as increases in sweating and heart rate.

Early psychophysiological research assumed that peripheral autonomic measures provided sensitive indicators of arousal. This view was based on a rudimentary understanding of the autonomic nervous system in which changes in electrodermal activity (e.g., sweating) and heart rate were assumed to be accurate indicators of sympathetic activity. As the activation arousal theory developed, a continuity between peripheral autonomic responses and central mechanisms was assumed (see Ref. [9], and sympathetic activity was assumed to parallel activation of the brain. According to this assumption, organs influenced by sympathetic efferent fibers, such as the sweat glands, blood vessels, or the heart were potential indicators of limbic or cortical activity [[10], [11], [12]].

Although the specific pathways relating these various levels were never outlined and are still sketchy, electrodermal (e.g., GSR) and heart rate became the primary focus of research during the early history of the Society for Psychophysiological Research. This was due to their presumed sympathetic innervation and, in part, to their measurement availability. By default, this emphasis created a research environment that neglected several important factors (a) parasympathetic (e.g., vagal): influences, (b) interactions between sympathetic and parasympathetic processes, (c) peripheral autonomic afferents, (d) central regulatory structures, (e) the adaptive and dynamic nature of the autonomic nervous system, and (f) phylogenetic and ontogenetic differences in structural organization and function. In the initial presentation Polyvagal Theory was an attempt to provide an integrated theory [2], based on the literatures of several disciplines, that would provide the basis for testable hypotheses relating autonomic function to sociality and health in humans and non-human mammals.

## 3 Evolution repurposes the neural regulation of the autonomic nervous system to support sociality

Evolution informed the development of Polyvagal Theory. Through the lens of evolution, the theory focused on how mammals adapted many of the phylogenetical ancestral structures that evolved to support survival in a hostile world. Note that the title of the initial publication presenting the theory [2] is actually a synopsis of the theory - O*rienting in a defensive world: Mammalian modifications of our evolutionary heritage. A Polyvagal Theory.* The title summarizes a phylogenetic narrative in which the survival of mammals was dependent on an ability to downregulate and modify the innate defensive systems that were inherited from their reptilian ancestors. These embedded vestigial circuits with their emergent adaptive strategies are embedded in genes of mammals. For mammals, whose survival is dependent on their sociality to cooperate, to connect, and to co-regulate (e.g. Ref. [5], the ancient defense programs had to be harnessed and repurposed to enable the expression of several defining features including signals of safety and calmness in proximity to another trusted mammal.

Polyvagal Theory's interest in investigating mammalian autonomic regulation from a phylogenetic perspective does not focus on the obvious similarities with more ancient vertebrates. Rather, it focuses on the unique modifications that enabled mammals to optimize their survival. Consistent with this theme, Polyvagal Theory focuses on the evolved neural circuits that enabled mammals to down regulate the sympathetic activation that could support mobilization to fight or flee, to reduce psychological and physical distance with conspecifics, and to functionally co-regulate physiological and behavioral state. The theory focuses on the transition from reptiles to mammals and emphasizes the neural adaptations that enable cues of safety to down regulate states of defense. Within Polyvagal Theory the evolutionary trend has led to a conceptualization of an emergent and uniquely mammalian social engagement system in which a modified branch of the vagus is integral. Neuroanatomically, this system is dependent on a brainstem area known as the ventral vagal complex. This area not only regulates the mammalian ventral cardio-inhibitory vagal pathway, but also regulates the special visceral efferent pathways controlling the striated muscles of the face and head. This does not preclude other structures being involved in mammalian social engagement behaviors or homologous structures in other vertebrates who do not share our phylogenetic history being involved in social engagement behaviors.

The relationship between mothers and their nursing offspring illustrates the social engagement system in action. To survive mammalian offspring must initially nurse as the primary mode of ingesting food. To nurse the infant must suck, a process dependent on a brainstem circuit involving the ventral vagal complex. Survival is dependent on the infant's nervous system efficiently and effectively coordinating suck-swallow-breathe-vocalize behaviors with vagal regulation of the heart through the ventral vagal pathways originating in the nucleus ambiguus. Through maturation and socialization, this ‘ingestive’ circuit provides the structural neural platform for sociality and co-regulation as major mediators to optimize homeostatic function leading to health, growth, and restoration (see Ref. [3]. For mammals there is a dependency between reactions to contextual cues and the function of this circuit. Cues of threat may disrupt, while cues of safety may enhance function. The sensory branches of the facial and trigeminal nerves provide major input into the ventral vagal complex. Functionally, changes in the state of this circuit, through the process of dissolution, will either ‘disinhibit’ phylogenetically older autonomic circuits to support defense (e.g., predator, disease, physical injury, etc.) or inform all aspects of the autonomic nervous system, including the enteric system (e.g. Refs. [13,14], to optimize homeostatic function.

Mammals uniquely have detached middle ear bones, which distinguishes mammals from reptiles in the fossil record. Detached middle ear bones expand the frequency band that mammals can hear and provide a ‘safe’ frequency bands in which they can socially communicate that will not be detected by reptiles.

Middle ear bones are small bones that separated from the jawbone during gestational development and form an ossicle chain that connects the eardrum to the inner ear. Small muscles regulated by branches of the trigeminal and facial nerves regulate the transfer function of the middle ear and determine the frequencies of sounds transduced through middle ear structures by controlling the stiffness of the ossicle chain. When the chain is stiff, the eardrum is tighter and low frequency sounds are attenuated, when the muscles relax, lower frequency sounds pass into the inner ear. In all mammalian species, based on the physics of their middle ear structures, there is a frequency band of perceptual advantage that is expressed when the middle ear muscles contract (see Ref. [15]. It is within this frequency band that social communication occurs, the low frequencies that through evolution been associated with predator are attenuated [16].

Interestingly, the coordination of the contraction and relaxation of these small muscles are frequently co-regulated with autonomic state and thus contract when there is strong ventral vagal tone to promote social communication and co-regulation. In contrast, when the autonomic nervous system shifts to a state of defense the muscles relax to detect low frequency predator sounds, which support defense strategies with auditory cues. The link between behavioral and autonomic state and listening is obvious in the study of language delays and auditory processing problems in children. Many children with problems in auditory processing also have behavioral state regulation limitations. This neurophysiological link provides a portal to regulate autonomic state through acoustic stimulation, which is easily observable when a mother calms her infant using prosodic vocalization. Similarly, we can observe the potent calming influences when a pet is calmed by the voice of a human. In addition, clinicians frequently report that survivors of trauma experience an auditory **hypersensitivity to background sounds and an auditory hyposensitivity to human voices** [17].

Through the evolution of vertebrates there are strong trends in the structures involved in regulating autonomic function. These trends may be summarized as moving from chemical to neural and then evolving greater specificity, efficiency, and speed through feedback circuits involving myelinated pathways. Evolution is a process of modification in which existing structures and circuits are modified to serve adaptive functions. In mammals, three primary autonomic states with specific neural circuits are observable and emerge at different times within the evolutionary history of vertebrates. In Polyvagal terms, the newest is labeled the *ventral vagal complex*, the oldest is *the dorsal vagal complex*, and in between is a spinal *sympathetic nervous system* evolved. Thus, evolution informs us of the sequence through which three circuits regulate autonomic function.

## 4 Dissolution explains responses to threat, chronic stress, and illness

Polyvagal Theory, following the work of John Hughlyns Jackson [1], assumes a phylogenetic hierarchy in which the newer circuits inhibit the older. Thus, when the ventral vagus and the social engagement system are dampened or go offline, the autonomic nervous systems move into a sympathetic state that supports mobilization. If this functional shift in state does not lead to a positive survival outcome, the autonomic nervous system may abruptly shut down via the dorsal vagal circuit. Jackson described this process of sequentially disinhibiting older structures as *dissolution* or evolution in reverse. Jackson used dissolution to explain the consequence of brain damage and disease, while Polyvagal Theory applies the principle of dissolution to adaptive autonomic reactions to cues of threat, which optimistically are reversible by cues of safety.

Convergent parallels with the phylogenetic evidence come from anatomical studies investigating human embryological origins and development of the autonomic nervous system through anatomical research via human autopsy studies (see Ref. [18]. Being informed by the autopsy studies, provides an opportunity to link maturational landmarks from anatomical studies with research monitoring fetal and preterm heart rate patterns. The neuroanatomical literature documents a maturational trend of myelination of the ventral vagus during the last trimester, with notable changes occurring after 30 weeks gestational age (see Ref. [3]. In our research we have been able to infer the maturation of the ventral vagal circuit by monitoring respiratory sinus arrhythmia in high-risk preterm newborns [[19], [20], [21], [22]].

In full term newborns it is possible to observe a predictable ‘dissolution’ sequence during challenging deliveries. This sequence is initiated by a withdrawal of ventral vagal tone (i.e., depressed respiratory sinus arrhythmia) leading to tachycardia and finally to potentially lethal bradycardia (see [33]). The literature supporting this maturational trend is summarized in Porges and Furman [3]. This literature documents that high-risk preterm newborns enter the post-partum world unprepared to cope with the environmental challenges. By entering the world with an immature ventral vagal circuit, the neural circuits regulating the high-risk newborn are prone to recruit the threat reactions of the functionally available sympathetic nervous system that would produce the metabolic costly tachycardia (i.e., increases in heart rate) or the dorsal vagal circuit that would produce a potentially lethal bradycardia (i.e., rapid and massive decreases in heart rate). Both reactions disrupt homeostatic function and compromise the preterm neonate's viability.

The survival challenges of the high-risk preterm infant provide a real-life example validating several features of Polyvagal Theory. First, we observe the hierarchical organization of the mammalian autonomic nervous system in the support of basic homeostatic processes that lead to optimized health, growth, and restoration. Juxtaposed to the traditional paired antagonism model of the autonomic nervous system that would focus on a hypothetical autonomic balance, we are informed that homeostasis requires the ventral vagus to functionally calm the autonomic nervous system to enable resources to be diverted from defense and directed towards health, growth, and restoration. Second, under challenge we observe the process of dissolution. Third, we observe the development and coordination of the social engagement system as the preterm develops the capacity to coordinate suck-swallow-breathe, which is followed by directed vocalizations and facial expressivity leading to sending cues of calmness and distress. These examples provide an understanding that stress or threat reactions may be operationally defined as the shifting of neural regulation of autonomic nervous system (i.e., withdrawal of ventral vagal tone) into a state that does not support homeostatic processes.

Metaphorically, the high-risk preterm neonate comes into the world with an autonomic nervous system that is more reptilian than mammalian. During the last trimester, the ventral vagus develops. Preterm neonates are frequently born prior to the development of a functional ventral vagus (see Refs. [21,22]. Without a functioning ventral vagus, the preterm neonate is predisposed to react with a ‘reptilian’ defensive response via the dorsal vagal pathway to stressors and challenges of birth. When recruited for defense, the dorsal vagal system may produce bradycardia and apnea. For reptiles, who are not as oxygen needy as mammals, this reaction supports survival by minimizing detection by the predator by appearing not to be living. However, for the oxygen needy mammals this response pattern is potentially lethal.

A preterm infant's depressed social engagement system will have a negative impact on caregivers, who are anticipating reciprocal cues of social connection via facial expressivity and prosodic vocalizations. The result may be a parent who feels they love their child, but their child does not love them. Similar disconnects have been felt, if not voiced, by parents who have children with dampened social engagement systems, such as children on the spectrum of autism. In addition, children of depressed parents may interpret their parents' dampened emotionality as reflecting disinterest or lack of love.

## 5 Polyvagal Theory: a testable solution of the vagal paradox

Polyvagal Theory emerged from my research studying heart rate patterns in human fetuses and newborns. In obstetrics and neonatology, the massive slowing of heart rate known as *bradycardia* is a clinical index of risk and assumed to be mediated by the vagus. During bradycardia, heart rate is so slow that it no longer provides sufficient oxygenated blood to the brain. This type of vagal influence on the fetal and neonatal heart could potentially be lethal. However, with the same clinical populations, a different index of vagal function was assumed to be a measure of resilience [21]. This measure was the respiratory oscillation in beat-to-beat heart rate variability (i.e., respiratory sinus arrhythmia), which was the focus of my research for several decades. Animal research demonstrated that both signals could be disrupted by severing the vagal pathways to the heart or via pharmacological blockade (i.e., atropine), interfering with the inhibitory action of the vagus on the sinoatrial node (for review see Ref. [2]. These observations posed a paradox. How could cardiac vagal tone be both a positive indicator of health when monitored with heart rate variability and a negative indicator of health when it manifests as bradycardia?

The resolution to the paradox came from understanding how the neural regulation of the autonomic nervous system changed during evolution and that the sequence of these phylogenetic shifts in global autonomic regulation was mirrored in prenatal development. The study of comparative neuroanatomy identified a structural change in vagal regulation occurring during the transition from primitive extinct reptiles to mammals. During this transition, mammals evolved a functional diaphragm (also observed in some reptiles) and a second cardio-inhibitory vagal motor pathway that originated in the ventral vagal nucleus (i.e., nucleus ambiguus). This vagal pathway provides the primary cardio-inhibitory influences on the heart. The circuit originating in the ventral vagal complex selectively services organs above the diaphragm (e.g., heart, bronchi) and interacts with the regulation of the striated muscles of the face and head via special visceral efferent pathways. This uniquely mammalian ventral vagal pathway is myelinated and conveys a respiratory rhythm to the heart's pacemaker, resulting in a rhythmic oscillation in heart rate at the frequency of spontaneous breathing known as *respiratory sinus arrhythmia.*

Polyvagal Theory uses evolution to highlight neuroanatomical and functional changes in how the brainstem regulates physiological state in mammals. These changes resulted in two vagal pathways operating in a hierarchal manner, with one pathway being protective and supporting homeostasis, while the other evolutionarily older pathway supports homeostasis only when the more evolutionarily modern vagus is functional [13]. Other than this coordinating role, this more modern vagal pathway has other attributes that functionally constrain, inhibit, and dampen other components of the autonomic nervous system that can be recruited in defense - the sympathetic nervous system that supports fight and flight behaviors and the older vagal circuit that triggers immobilization, behavioral shutdown, diarrhea, and potentially lethal bradycardia. Thus, when the newer circuit is withdrawn (i.e., dissolution) there is a shift in physiological state (i.e., loss of ventral vagal tone) with major survival-related consequences as the neural regulation of the autonomic nervous system shifts to defensive strategies from coordinating other attributes of the autonomic nervous system that optimize health, growth, restoration, and social behavior.

Polyvagal Theory extracts from contemporary neuroanatomy, neurophysiology, and evolutionary biology several basic uncontroversial conclusions: 1) mammals have two vagal pathways (i.e., supra-diaphragmatic, subdiaphragmatic), 2) evolution and development provide insight into the changes in brainstem structures that enable mammals to be physiologically calm and socially interact. Functionally, mammals have neural attributes that act efficiently via rapidly responding cardioinhibitory fibers (e.g., ventral vagal pathways), capable of calming to promote social communication. The ventral vagal pathways also coordinate and repurpose circuits that evolved to support defense in socially relevant processes, such as play (i.e., ventral vagal influences constrain sympathetic reactivity), and intimacy (i.e., ventral vagal influences constrain dorsal vagal reactivity).

## 6 Polyvagal Theory links the mammalian autonomic nervous system to sociality

The polyvagal model emphasizes the evolutionary transition from the extinct reptiles to primitive mammals to modern mammals and humans. This transition resulted in the capacity to functionally ‘retune’ the autonomic nervous system, thus fostering social engagement behaviors and permitting physiological state co-regulation through social interactions. The theory also provides an understanding of increased metabolic demands of mammals, when compared to reptiles. The theory emphasizes the need for social interactions in regulating the human autonomic nervous system and in fostering homeostatic functions. The theory further emphasizes that there are unique attributes of the mammalian autonomic nervous system that differ from reptiles and other earlier vertebrates including the integration of brainstem structures (i.e., ventral vagal complex) to coordinate the regulation of the ventral vagal nucleus (i.e., nucleus ambiguus) with special visceral efferent pathways emerging from cranial nerves V, VII, IX, X, and XI to form an early developmental survival-related suck-swallow-breathe-vocalize circuit. As these pathways mature, they form, as proposed by the Polyvagal Theory, a spontaneous social engagement system that supports homeostasis and co-regulation.

This link between vagal activity and social engagement behaviors potentially can be monitored through the output of ventral vagal pathways on the heart, providing a diagnostic and prognostic index of cardiac vagal tone (i.e., respiratory sinus arrhythmia). It is important to emphasize that respiratory sinus arrhythmia, by providing a quantifiable portal of the vagal contribution to the Social Engagement System, enables the structuring of testable hypotheses related to Polyvagal Theory. However, the theory is not dependent on the attributes or even the evolutionary history of respiratory sinus arrhythmia. Thus, the theory links mental and physical health and well-being through the co-regulation of autonomic state through social behavior. Since the model is based on a detailed literature review, the reader is encouraged to read the initial presentation of the theory (see Ref. [2], and the subsequent updates [7,23,24].

## 7 Autonomic state as an intervening variable

Central to Polyvagal Theory is the role that the autonomic nervous system plays as an intervening variable influencing mammalian behavioral and physiological reactions to challenges both in the body (e.g., illness and distress) and in the environment (e.g., cues of threat and safety). The theory encourages us to think of autonomic state as a functional neural platform from which different adaptive behaviors and reactions may spontaneously emerge. The theory embraces a stimulus-organism-response (S-O-R), with autonomic state being a measurable intervening variable, rather than a deterministic S-R model that has been prevalent in both behaviorally oriented psychology and mechanistic models of physiology. The theory proposes that there are three global autonomic states that functioned, in general, as a hierarchy that are phylogenetically ordered. In this model, the evolutionarily newest vertebrate autonomic modification is a mammalian ventral vagal circuit with features that support the mammalian dependence on transporting oxygenated blood to the brain and visceral organs and the regulation of physiological state through sociality and ingestion.

## 8 Neuroception

Polyvagal Theory proposes that the neural evaluation of risk and safety reflexively triggers shifts in autonomic state without requiring conscious awareness. Thus, the term “neuroception” was introduced to emphasize a neural process, distinct from perception, capable of distinguishing environmental and visceral features that are safe, dangerous, or life-threatening (Porges, 2003, 2004). A form of neuroception can be found in virtually all living organisms, regardless of the development of the nervous system. In fact, it could be argued that single celled organisms and even plants have a primordial nervous system that respond to threat. As mammals, we are familiar with reactions to pain (i.e., nocioception), a type of neuroception. We react to pain prior to our ability to identify the source of the stimulus or even of an awareness of the injury. The detection of threat appears to be common across all vertebrate species. However, mammals have an expanded neuroception capacity in which they not only react instantaneously to threat, but also respond instantaneously to cues of safety. It is this latter feature that enables mammals to down regulate defensive strategies to promote sociality by enabling psychological and physical proximity without the consequences of injury. It is this calming mechanism that adaptively adjusts the central regulation of autonomic function to dampen sympathetic activation and to protect the oxygen-dependent central nervous system, especially the cortex, from the metabolically conservative reactions of the dorsal vagal complex (e.g., fainting, death feigning).

Polyvagal Theory proposes that neuroception functionally involves both top-down and bottom-up mechanisms. The process is assumed to be initiated via top-down pathways involving cortical areas located in or near temporal cortex that reflexively interpret cues of threat and safety. These areas of the cortex are sensitive to the intentionality of biological movements including voices, faces, gestures, and hand movements. Embedded in the construct of neuroception is the capacity of the nervous system to react to the intention of these movements. Neuroception functionally decodes and interprets the assumed goal of movements and sounds of inanimate and living objects. Thus, the neuroception of familiar individuals and individuals with appropriately prosodic voices and warm, expressive faces frequently translates into a positive social interaction, promoting a sense of safety. Autonomic state responds to the top-down detect of risk or safety. The autonomic reactions sends sensory information regarding bodily feelings to the brain where they are interpreted and consciously felt. The bottom-up limb of the neuroception is functionally equivalent to interoception. Thus, although we are often unaware of the stimuli that trigger different neuroception responses, we are generally aware of our body's reactions (i.e., visceral feelings) embodied in other autonomic signatures that support adaptive behaviors (i.e., social engagement, fight/flight, shutdown).

## 9 Polyvagal Theory leads to testable hypotheses and potential interventions

Once the conceptualization of the vagal brake was introduced within the Polyvagal Theory (Porges, 1996), the commonly observed changes in global measures of heart rate variability and the more specific ‘vagal’ component of respiratory sinus arrhythmia during psychological and physical challenges could be understood from a neurobiological perspective. This perspective has embraced technologies to quantify specific neural signals such as respiratory sinus arrhythmia as an accurate index of ventral vagal tone [16]. Similarly, once the integrated Social Engagement System [23] was introduced into the theory, then the cues of safety and trust that are features of social support could be mechanistically understood as supporting greater vagal regulation and healthful homeostatic functions.

We live in a culture that honors technologies as potential interventions to optimize mental and physical health. The proliferation of various noninvasive trigeminal and vagal nerve stimulators, work through feedback mechanisms described in the Polyvagal Theory. Consistent with polyvagal principles, these devices stimulate afferent pathways that go to the brainstem area that communicate with the ventral vagus to increase vagal outflow resulting in calming and optimized homeostatic function. As we are informed by neuroanatomy and the phylogenetic development of an integrated Social Engagement System, we learn that listening is an alternative portal to regulate ventral vagal tone and the entire Social Engagement System.

Recently I developed an intervention called the Safe and Sound Protocol that uses computer altered modulated vocal music to amplify the prosodic cues of social communication (see Refs. [25,26] These are similar to the acoustic cues that human and other mammalian nervous systems interpret as cues of safety and trust. For example, a mother's vocal cues have the capacity to signal safety and calm her child (e.g., infant directed speech) and vocal cues by adults to calm their children and pets. These acoustic cues increase neural tone to middle ear muscles, which are regulated by brainstem structures (see Refs. [15,16] involved in responding to threat and safety similar to the vagal brake. Under threat the middle ear muscles relax and the eardrums become sensitive to low frequency sounds to detect movements that could be potentially dangerous. In this state, the ability to detect human speech may be compromised. When there are cues of safety, the body calms and the autonomic nervous system is in a state predominantly regulated by the ventral vagus. This ventral vagal state is also reflected in a more melodic or prosodic human voice via a non-cardiac branch of the vagus, the recurrent laryngeal nerve. Thus, the auditory system is an important portal to our autonomic nervous system. Functionally the intervention leverages an understanding of neuroception and the cues that trigger a state of increased ventral vagal tone observed in behaviors of calmness, accessibility, and spontaneous social engagement. When the initial patent [27] was awarded for the technologies embedded in the Safe and Sound Protocol, the specific claim that the acoustic stimulation functioned as an acoustic vagal nerve stimulator was accepted.

## 10 What is Polyvagal Theory?

Polyvagal Theory focuses on differences, rather than similarities between vertebrate species and especially the neuroanatomical and functional phylogenetic changes that we observed as a product of the transition from asocial reptiles to social mammals. For example, we see profound distinctions between the mammalian respiratory sinus arrhythmia and heart rate-respiratory interactions in other vertebrates. In mammals there is a well-defined common central respiratory oscillator that sends a respiratory rhythm from the brainstem to both the heart and the bronchi. This information flows through the vagal neurons originating in the nucleus ambiguus. In fact, the oscillator can be conceptualized as an emergent property of the interactions among structures regulated by the nucleus ambiguus including the larynx and pharynx [28]. This, of course, is not consistent with the features of a primitive nucleus ambiguus that might be seen in vertebrates that preceded mammals. The observation of a common central oscillator is functionally unique to mammals and is the neurophysiological foundation enabling the quantification of respiratory sinus arrhythmia to function as a portal to measure ventral vagal tone.

As a theory, Polyvagal Theory has two components: 1) a descriptive model, and 2) a series of hypotheses related to explanations and applications. The first component is a model of the mammalian autonomic nervous system. The second component is hypothesis driven and future oriented, which could potentially lead to enhancements of mental and physical health. This chapter was written to help researchers distinguish between the interdisciplinary literature that supports the principles of the theory and the testing of hypotheses derived from the theory. The interdisciplinary literature supports a convergence among sources of evidence including the literatures describing: 1) the development of the neural structures regulating the autonomic nervous system, 2) the insights derived from studying the phylogeny of the autonomic nervous system, 3) research evaluating autonomic reactions to chronic stress and threat, and 4) clinical observations illustrating the principle of dissolution in physical and mental illness especially in studying both high-risk preterm infants and the personal histories of individuals who have survived trauma.

As our science evolves hopefully new technologies will be developed to measure the dynamic regulation of the dorsal vagus. When this is accomplished then a Polyvagal-informed autonomic mapping of an individual could potentially evaluate the function of each pathway. Currently, although dependent on an accurate metric of the ventral vagal tone, we have discovered a new dimension of this system that reflects ‘vagal efficiency.’ Vagal efficiency is a metric that evaluates the effectiveness of dynamic change in the ‘vagal brake’ (i.e., changes in the amplitude of respiratory sinus arrhythmia) on dynamic heart rate. In our initial published research, we identified sleep state differences in vagal efficiency in newborns [29], a maturational influence on vagal efficiency in high-risk preterm infants [22], a sensitivity to alcohol [34] and the utility of vagal efficiency in predicting pain reduction during noninvasive vagal stimulation in adolescents with functional abdominal pain disorders (Kovacic et al., 2020). In our ongoing research, we are observing that individuals with features of dysautonomia had noticeable low vagal efficiency when compared to healthy controls [30]. In addition, in another study we noted low vagal efficiency in survivors of trauma and maltreatment. Within the concept of dissolution, a reduction in vagal efficiency may reflect a dampening of feedback involved in regulating autonomic function that may precede end organ dysfunction.

## 11 How would research test attributes of the Polyvagal Theory?

The Polyvagal Theory emphasizes the role of autonomic state as an intervening variable in how we respond to internal and external cues. The theory changes the personal narrative from a documentary of events to a personalized narrative of feelings (i.e., autonomic state). Applications of Polyvagal Theory in the clinical world focus on autonomic state as a mediator of mental and physical health problems. For example, trauma retunes the autonomic nervous system from calmness and spontaneous social engagement to defense, thus interfering with the ability to socially engage, communicate, and connect. By placing autonomic state in the model as an intervening variable, neural regulation of the autonomic nervous system becomes both an assessment of neural state that would promote safety or defense and a portal for intervention. Functionally, **calm physiological states become more common and aberrant behaviors become less prevalent.**

The theory was not proposed to be either “proven” or “falsified,” but rather to be informed by research and modified. Claims of falsification would argue that the evolved changes in the autonomic nervous system would not support a social engagement system and that socially delivered cues of safety would not calm the autonomic nervous system and consequentially socialize behavior. The theory is dependent on evolution and development to structure a hierarchical model of autonomic function inclusive of the Jacksonian principle of dissolution. This model could explain how co-regulatory social interactions are not merely ‘social’ behaviors, but neuromodulators of autonomic state via an integrated social engagement system that is capable of either supporting or disrupting homeostatic functions. Thus, aspects of social behavior can functionally support or disrupt health.

The theory uses evolution to extract a phylogenetic sequence of autonomic regulation. This sequence identifies stages during vertebrate evolution when a spinal sympathetic nervous system and the two vagal pathways emerged and become functional via maturation in mammals. It would be difficult to argue that the sequence does not occur, although it would be possible to identify antecedent similarities in most vertebrates regardless of class or group. **The question is not whether there are similarities in ancestral vertebrates, but rather how have these circuits been adapted to provide a unique mammalian autonomic nervous system that is intimately intertwined with co-regulatory social behavior.**

There are many examples of the unique type of sociality expressed in mammals, which differentiates mammals from their reptilian ancestors. Since Polyvagal Theory is focused on the phylogenetic transition from reptiles to mammals, it does not focus on other the sociality of other vertebrate species that evolved from reptiles (e.g., birds) following the divergence of mammals. Polyvagal Theory is dependent on the processes that evolutionary theorists describe as ex-adaptation and co-opting. These processes involve modifications that shift in the function of a structure during evolution. For example, a structure can evolve because it served one particular function, but subsequently it may come to serve another. Ex-adaptation and co-opting are common strategies of repurposing vestigial structures in both anatomy and behavior. Polyvagal Theory is agnostic about the evolutionary pressures that result in the selection of specific changes. In contrast, the theory is more ‘phylogenetically’ descriptive and more focused on the functional outcome of repurposing. More specifically, the theory is interested in how the structures regulated by the ventral vagal complex were repurposed to provide the regulation of an integrated social engagement system that provide the primary portal to socially engage and communicate, ingest, and calm. Current neuroanatomical knowledge documents the refinement of the ventral vagal complex in mammals. Thus, although the ventral vagus (i.e., originating in the nucleus ambiguus) may have an origin in reptiles [31], it appears that it is only in mammals that this pathway has been repurposed to convey and respond to social cues, via neuroception, as a potent mediator of autonomic state.

## 12 Concluding comments

Evolution transformed attributes of the autonomic nervous system into an integrated social engagement system that incorporated a brainstem communication area (i.e., the ventral vagal complex) that regulated, via special visceral efferent pathways, the striated muscles of the face and head and coordinated these processes with the vagal regulation of the heart and the bronchi. In mammals the ventral vagal complex enables the coordination of a suck-swallow-breathe-vocalize system with the vagal regulation of the heart. As the neuroanatomy of this ingestive circuit matures, the circuit becomes a functional social engagement system that enables physiological state to be communicated to conspecifics via facial expression and vocalizations. This does not preclude the validity of observations that document in earlier vertebrates links between the brainstem regulation of the special visceral efferent and the source nuclei of the vagus. For example, in more primitive vertebrates the special visceral efferent pathways regulate structures that evolved from ancient gill arches. Thus, in fish there is often a synchrony between gill movements and heart rate, although the regulation of heart rate is mediated through the dorsal vagal nucleus. However, in mammals these structures and their neural regulation have been modified via evolution to support functions unique to the survival of mammals such as nursing and social communication via an integrated social engagement system dependent on the ventral vagal complex. This transition in neuroanatomy and function provides the basis to understand that for humans, similar to other mammals, connectedness and trusting relationships, are direct expressions of our biological imperative and are integrated into our biology.

## Funding

This work was supported by gifts to the Traumatic Stress Research Consortium from the Dillon Fund, Chaja Stiftung, and the United States Association for Body Psychotherapy.

## Declaration of competing interest

This work was supported by gifts to the Traumatic Stress Research Consortium from the Dillon Foundation, Chaja Foundation, and the United States Association of Body Psychotherapy.
